# Pharmacology: The Pharmacodynamics of Nutrients and Nutrient Interactions in Biological Functions

**DOI:** 10.1155/2015/974572

**Published:** 2015-12-22

**Authors:** M. Hasan Mohajeri, Gunter P. Eckert, James R. Pauly, Christopher M. Butt

**Affiliations:** ^1^DSM Nutritional Products, 4303 Kaiseraugst, Switzerland; ^2^Goethe University, 60438 Frankfurt, Germany; ^3^University of Kentucky, Lexington, KY 40536, USA; ^4^DSM Nutritional Products, Boulder, CO 80301, USA

Epidemiological studies and randomized controlled trials (RCTs) have shown that nutrition and nutritional habits may play a critical role in the optimal functioning of biological systems from conception to old age [1]. Epidemiological studies, due to their methodology, can only provide correlations between consumption of nutrient(s) and biological outcomes, whereas RCTs normally study just one dose of a certain nutrient. Both study types are therefore ill-suited to study the mechanisms by which nutrients exert their benefits. Moreover, the nutrients' functions may depend on each other. For example, B-vitamins' functions are known to be interdependent [2]. While the exact mechanisms are unclear, the course and severity of conditions such as obesity, cellular aging, cancer, and neurological disorders can be affected by nutritional approaches [3]. Thus, food and nutrition play an intimate and inextricable role in human health. Despite growing interest in adequate nutrition, the effects of nutrient interaction, the possible varying effects on different organs, and the dependency of such effects on age or health status are complicated topics that deserve careful examination.

The pharmacodynamics of nutrients, or the so-called nutridynamics, is the term used to describe how a food component is affected by other food ingredients and what a particular ingredient does in the body [4]. The intention is to systematically study the mechanism of action, that is, how an effect is produced. Obviously, this is a challenging task due to presence of manifold targets and the involvement of multiple biological systems for each nutrient. As an example, depending on the available amount of a given micronutrient, some biological functions may be put on hold, so that the limiting micronutrient can be used for biological functions that are indispensable to life [5].

This special issue presents several research articles studying a few selected nutrients and their function in human biology by* in vitro*,* in vivo,* and human experiments. E. Adamska et al. aimed to determine the metabolic response after intake of standardized meals with various fat and carbohydrate contents and to determine the differences among normal-weight and overweight/obese men. Glucose, insulin, triglyceride, and free fatty acid levels were measured at fasting state and 30, 60, 120, 180, and 240 minutes after meal intake. The results showed that the postprandial response depended on both the meal macronutrient content and the body mass index (BMI). In another study, R. A. O. Cruz and colleagues compared the antioxidant activity of sorghum kafirin and sorghum flour and their influence on lipids and antioxidant capacity in rats. Sorghum is a grain that has a high content of fiber, protein, mineral, and polyphenols. *α*-kafirin is the main storage protein in sorghum [6]. Rats supplemented with sorghum kafirin extract exhibited improved lipid metabolism and increased serum antioxidant potential, especially when cholesterol was added to the feed. These data may suggest that sorghum kafirin extract may be beneficial against atherosclerotic conditions. The effects of nutritional intervention on markers of oxidative stress were also studied in large domestic animals. C. Tan et al., supplemented oregano essential oil (OEO) to the diets of 60 white sows during gestation and lactation. Oxidative stress status, colostrum and milk composition, lactation feed intake of sows, and piglet growth performance were determined. The OEO supplementation led to a dramatic reduction of serum levels of several reactive oxygen species at all ages studied and improved piglet growth, which was attributed to the reduction in oxidative stress.

In two* in vitro* studies, G. Ravacci et al. and G. La Fata et al. set out to examine the roles of docosahexaenoic acid (DHA) and vitamin E in cellular models related to breast cancer and aging, respectively. The hypothesis that lipid metabolism may stimulate malignancy in breast cancer was tested by overexpressing the HER2 proto-oncogene (human epidermal growth factor receptor 2) in a normal breast cell line. Overexpressing and normal cells were then treated with trastuzumab (interfering monoclonal antibody) and DHA. The data provided good evidence that the oncogenic transformation of breast cells by* HER2* overexpression may require a reprogramming of lipid metabolism that is independent of the mTORC1 pathway and peroxisome proliferator-activated receptor gamma (PPAR*γ*) activity. Moreover, DHA inhibited the reprogramming of cancerous cells. Last, but not least, it was shown by using an unbiased automated quantification method in two different human primary cell types that acute or chronic vitamin E treatments may slow down cellular senescence. Mechanistically, this antiaging vitamin E effect could be due to downregulation of the expression of the cyclin-dependent kinase inhibitor P21. Further studies are warranted to clarify whether this effect is dependent on antioxidative properties of vitamin E or whether this possible antiaging affect belongs to new emerging functions of vitamin E which go beyond the antioxidative function.

In summary, the interaction of food ingredients may affect their biological functions as well as the extent of their benefits on growth, repair, and maintenance of biological systems. These areas of research need to be intensified in order to develop meaningful recommendations of dietary guidelines to the general public and/or to individuals with specific needs.

*M. Hasan Mohajeri* *Gunter P. Eckert* *James R. Pauly* *Christopher M. Butt*
